# Circular RNAs in Pregnancy and the Placenta

**DOI:** 10.3390/ijms23094551

**Published:** 2022-04-20

**Authors:** Anya L. Arthurs, Tanja Jankovic-Karasoulos, Melanie D. Smith, Claire T. Roberts

**Affiliations:** Flinders Health and Medical Research Institute, Flinders University, Bedford Park, SA 5042, Australia; tanja.jankovickarasoulos@flinders.edu.au (T.J.-K.); melanie.smith@flinders.edu.au (M.D.S.)

**Keywords:** circular RNA, circRNA, pregnancy, reproduction, placenta

## Abstract

The emerging field of circular RNAs (circRNAs) has identified their novel roles in the development and function of many cancers and inspired the interest of many researchers. circRNAs are also found throughout the healthy body, as well as in other pathological states, but while research into the function and abundance of circRNAs has progressed, our overall understanding of these molecules remains primitive. Importantly, recent studies are elucidating new roles for circRNAs in pregnancy, particularly in the placenta. Given that many of the genes responsible for circRNA production in cancer are also highly expressed in the placenta, it is likely that the same genes act in the production of circRNAs in the placenta. Furthermore, placental development can be referred to as ‘controlled cancer’, as it shares many key signalling pathways and hallmarks with tumour growth and metastasis. Hence, the roles of circRNAs in this field are important to study with respect to pregnancy success but also may provide novel insights for cancer progression. This review illuminates the known roles of circRNAs in pregnancy and the placenta, as well as demonstrating differential placental expressions of circRNAs between complicated and uncomplicated pregnancies.

## 1. Introduction

The placenta, a product of conception with a transient existence, uniquely supports pregnancy. It plays a critical role in nutrient, waste and gas exchange between the mother and fetus. Correct placentation underpins fetal development, as well as coordinating maternal adaptations to pregnancy to maintain maternal and fetal health. In pregnancy complications characterised by aberrant placentation such as preeclampsia (PE) [1] and intrauterine growth restriction [2], there is an altered placental transcriptome. Emerging evidence demonstrates the roles of novel RNA species in pregnancy complications, particularly circular RNAs (circRNAs).

The first identified circRNA, the hepatitis D viroid, was reported in 1977 [3]. Following this, circRNAs were found in mammalian cells using electron microscopy in 1979 [4]. Initially, due to their low abundance, they were disregarded as products of misplicing. Then, in 1991, circular transcripts were found in a variety of normal and neoplastic human cells [5]. As technology improved, primarily in sequencing capabilities and bioinformatics, so has the study of the structure and functionality of circRNAs [6].

Many circRNAs have evaded detection until now for two main reasons. Unlike other small RNAs, circRNAs are not able to be easily separated from mRNA through size fractionation or electrophoretic mobility as often they differ from their linear form only in circular structure. They are also easily destroyed by molecular techniques requiring amplification or fractionation due to their circular form and, as they lack polyadenylation, they are often discarded when analysing sequencing data [7]. These covalently closed circular RNA structures remain enigmatic, with a plethora of reported functions and methods of biogenesis. This review will detail what is currently known about circRNAs, their implications for placental development and function, and their broader consequences for pregnancy.

## 2. circRNA Biogenesis

**Figure 1:**
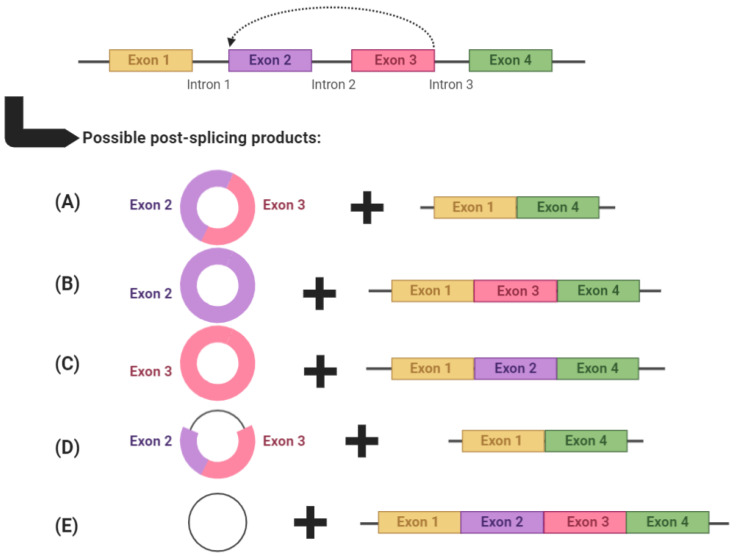
An example of circRNA biogenesis using backsplicing. circRNAs are produced through backsplicing and can potentially produce several alternatively spliced products. circRNAs can: (**A**) comprise multiple exons, (**B**,**C**) comprise a single exon, (**D**) comprise both exons and introns (termed exonic intronic circRNAs) or (**E**) comprise only introns (termed circular intronic RNAs). After each backsplicing event, the remaining exons are left to form an alternatively spliced transcript. Graphic created with BioRender.com, accessed on 5 May 2021.

circRNAs are produced through backsplicing, a process in which the 3′ end of a downstream exon is spliced and covalently linked with the 5′ end of an upstream exon [8,9,10]. This often leaves behind a transcript which becomes an alternatively spliced linear RNA product with skipped exons (Figure 1) [11]. The end circRNA product is devoid of 5′ capping and 3′ polyadenylation, and is consequently resistant to exonuclease activity [12]. It is possible for circRNAs to consist of one or more exons, sometimes including introns [termed exonic intronic circRNAs (EIcircRNAs)], or even introns only [termed circular intronic RNAs (ciRNAs)]. Exonic circRNAs make up approximately 80% of circRNA transcripts and are mainly located in the cytoplasm, whereas EIcircRNAs and ciRNAs tend to be located in the nucleus and regulate their cognate linear transcripts [13].

It has been shown that the biogenesis of circRNAs, while via backsplicing, still involves canonical splicing signals and spliceosomal mechanisms [12]. The experimental use of isoginkgetin, a splicing inhibitor, inhibits the formation of circRNAs, as well as linear RNAs [14,15]. Moreover, mutations in the canonical splicing sites of exons inhibit circularisation and circRNA biogenesis [16,17,18]. The biogenesis of circRNAs is in constant competition with linear RNA production through canonical splicing machinery [15]. It has been shown that the elongation velocity of RNA polymerase II positively correlates with backsplicing efficiency [19]. This has been corroborated by several studies in which mutations in the RNA polymerase II large subunit significantly reduced RNA pol II elongation velocity, and thus, backsplicing efficiency and circRNA production [20,21,22].

**Figure 2:**
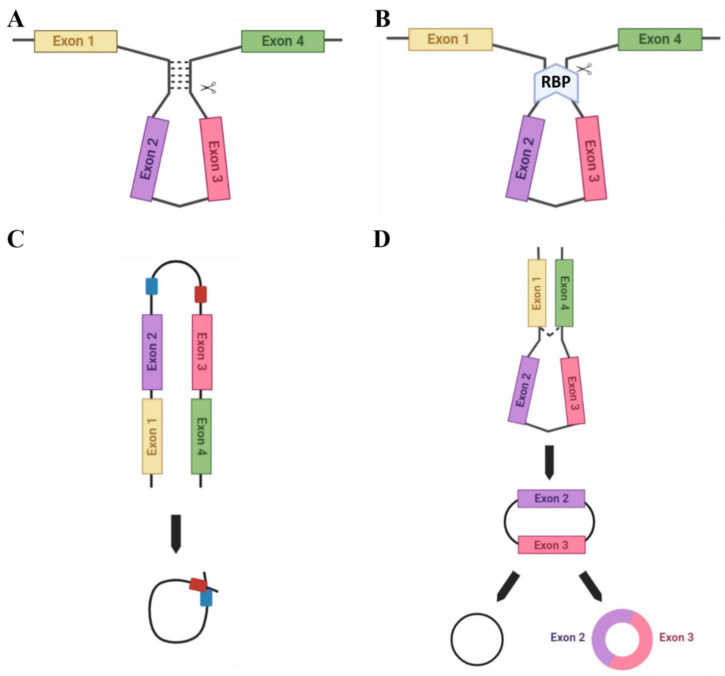
Methods of circRNA biogenesis. A number of different methods facilitate the circularisation of circRNAs: (**A**) Complementary base-pairing (e.g., via Alu repeats) promotes backsplicing due to spatial reduction in the splice sites. (**B**) RBP-driven circularisation occurs when RBPs bind flanking introns and bridge them together for splicing. (**C**) ciRNA formation: ciRNAs are formed from lariat introns that escape debranching. C-rich (red) and GU-rich (blue) sequence binding is sufficient for the intron to avoid debranching and generate a ciRNA. (**D**) The lariat-driven model of circularisation. Exon-skipping occurs to bring splice sites into close proximity.

There are several ways in which circRNAs can be produced (Figure 2). Complementary base-pairing (Figure 2A) occurs when complementary inverted sequences in introns flanking backsplice junctions facilitate circularisation by base-pairing to form a stem-loop-like structure which can then be cleaved to form a circRNA. This structure promotes spatial reduction in splice signals required for backsplicing and thus contributes to RNA circularization [23,24]. Specifically, Jeck et al. [25] first reported on the importance of inverted ALU repeat elements in backsplice-flanking introns in facilitating circRNA biogenesis. ALU repeats are short nucleotide sequence repeats that comprise approximately 11% of the genome and are primate-specific [26]. These inverted ALU repeats are five times more enriched in sites of human exonic circRNAs formation. There are also examples of exonic circRNAs where the entire gene is circularised and no upstream or downstream exons are leftover for alternatively spliced transcript production, such as *SRY*, the male sex-determining gene found on the Y chromosome, which abundantly produces circRNAs [27].

Certain RNA binding proteins (RBPs) are also able to facilitate RNA circularisation (Figure 2B). For example, the RBP Quaking (QKI), which is highly expressed in the placenta, aids the biogenesis of circRNAs which are involved in epithelial-mesenchymal transition (EMT), a process common to placental development and many cancers. Furthermore, QKI knockdown subsequently inhibits the production of EMT-related circRNAs [18]. However, for correct functioning QKI requires the assistance of binding sites in introns flanking the exons to facilitate circRNA biogenesis [18]. Alternatively, the RBP Muscleblind (MBL), which is also highly expressed in the placenta, facilitates the biogenesis of the circRNA (circMbl) from its own cognate RNA by binding to specific MBL conserved sites in flanking introns [15].

circRNAs can also be formed from RNA lariats (lasso-shaped by-products of RNA splicing), termed circular intronic RNAs (ciRNAs). Distinct from exonic circRNAs, which feature a 3′-5′ carbon linkage at the splicing branchpoint, lariat RNAs feature 2′-5′ linkages [23]. They can be formed utilising a consensus motif with a GU-rich region, located near the 5′ splicing point, and a C-rich region, near the branchpoint site in ciRNA-producing introns, which allow for intron lariat escape from debranching. These regions then facilitate the circularisation of this intron [28,29] (Figure 2C) and the 3′ ‘tail’ downstream from the branch point is trimmed to stabilise the ciRNA and protect from exonucleases. This motif is not enriched in regular introns [23] and has been suggested as an essential RNA element to expedite intron lariat escape from debranching.

The lariat-driven model of circularisation (Figure 2D) can encompass a variety of the above techniques for circRNA biogenesis. Middle exons of a linear transcript are ‘skipped’ to allow an upstream 3′ splice donor to covalently bond to a downstream 5′ splice acceptor. The spliceosome then removes the introns to form the final circRNA product.

## 3. circRNA Function

**Figure 3:**
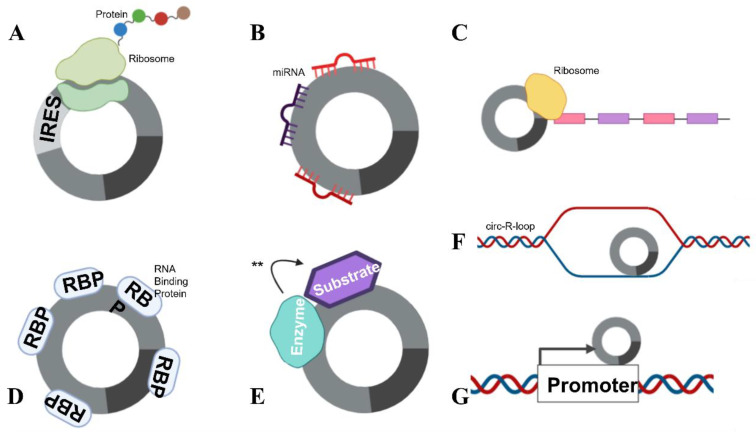
circRNA functions. circRNAs are able to complete numerous roles: (**A**) Translation can occur in the presence of an IRES. (**B**) circRNAs can function as miRNA sponges, by “mopping up” miRNAs and preventing their actions. (**C**) mRNA traps (inhibits translation) by sequestering the translation start site on mRNA. (**D**) circRNAs are also able to bind proteins, including RBPs and (**E**) enzyme–substrate complexes, to facilitate actions (denoted with **) such as phosphorylation, ubiquitylation and acetylation. (**F**) circRNAs can form circ-R-loops with DNA and impede transcription, facilitating DNA breaks. (**G**) circRNAs can also influence the host promoter region, altering DNA replication and transcription.

There are several functions for circRNAs that have been identified to date. A small number of circRNAs are able to be translated (Figure 3A) (e.g., the Hepatitis δ agent, a circular RNA satellite virus of the Hepatitis B virus [30]) while engineered circRNAs can undergo translation if an internal ribosomal entry site (IRES) is included in the design [7]. However, the majority of circRNAs appear to be non-coding.

Some specific, highly expressed circRNAs function as miRNA sponges (Figure 3B). The exonic circRNAs from *CDR1as* [31], cerebellum-related antigen 1, and *SRY* [12], the testis-determining factor, have been shown to bind miRNAs without degrading them, inhibiting their function. Each of these circRNAs also has multiple miRNA binding sites in its sequence. The circRNA for *CDR1as* has 74 confirmed sites for miR-7 binding, as well as being densely seeded with Argonaute protein binding sites which allow for Argonaute–miRNA complexes to bind. The circRNA for *SRY* has 16 binding sites for miR-138 and coprecipitates with Argonaute 2. However, the concept that circRNAs act as miRNA sponges has recently been debated.

Whilst it is true that some circRNAs function efficiently as miRNA sponges, such as ciRS-7 [12], the notion of circRNAs functioning as sponges has been questioned due to the stoichiometric ratio of circRNA to miRNA molecules within the cell [32]. Given that the majority of circRNAs are produced at less than 2.5 copies per cell [18], it is improbable that they are able to significantly regulate the expression of miRNAs, which are often produced at 900–80,000 copies per cell [33]. The potential for circRNAs to mediate miRNA expression is likely to be reserved only for circRNAs with unusually high expression within cells, and multiple miRNA binding sites per molecule. Thus, new studies to examine the potential function of circRNAs as miRNA sponges may need to seek further validation through experiments that involve more than dual luciferase assays. However, this is not to suggest that many circRNAs do not have important cellular functions. As the majority of circRNAs are produced at ~2.5 copies per cell, this indicates an approximate 1:1 ratio with the DNA transcripts in each cell. Indeed, interaction with DNA is another function of circRNAs that has important implications in molecular biology (this is explored further below).

circRNAs can also function as transcriptional regulators, termed “mRNA traps” (Figure 3C). One example of this is the exonic circRNA produced from the *Fmn* (flavin mononucleotide) gene in mice, which is proposed to sequester the translation start site on the mRNA, reducing protein synthesis [34]. circRNAs can also bind proteins (Figure 3D), as previously mentioned, circMbl can sequester the Muscleblind RBP [15]. Furthermore, circANRIL, a circRNA in the antisense non-coding RNA in the INK4 locus (ANRIL) long non-coding RNA, regulates the maturation of precursor ribosomal RNA, therefore controlling ribosome biogenesis [15]. circRNAs have also been shown to facilitate the phosphorylation, ubiquitylation and acetylation [15] of proteins (Figure 3E), and participate as structural components of protein complexes [15]. Importantly, circRNAs have been shown to bind to, and facilitate breakages in DNA (see below) (Figure 3F). circRNAs have also been reported to recruit proteins to specific subcellular loci [15] and influence host transcript promoter regions (Figure 3G). With their many attributed functions, it is no surprise that circRNAs have been implicated to play a role in many pathophysiological and physiological states; this review will focus on their role in pregnancy.

## 4. The Role of circRNAs in Pregnancy

circRNAs are expressed throughout reproductive tissues in healthy pregnancy and are differentially expressed between healthy and complicated pregnancy. However, the question of whether this is cause or effect requires further research. Studies that have examined circRNAs expressed in reproductive tissues and detected in maternal serum in pregnancy are limited and summarised in Table 1.

In animals, research conducted in murine models has described circRNA profiles in oocytes and pre-implantation embryos [35] and in both implantation and inter-implantation sites in the endometrium [41]. The sizable differences in the profiles between these cells and tissues indicate that circRNAs play a role in the reproductive process. Interestingly, one study was completed in both in vitro cell lines and in vivo rat experiments to demonstrate the effect of circSFXN1 (sideroflexin 1) in PE pathology [53]. sFLT1-expressing adenovirus injections into rats induced a PE-like phenotype, which was abated by treatments with si-circSFXN1. This clearly demonstrates the pathological potential for aberrant circRNA expression. Another study examined atretic follicles in porcine ovaries, determining that a circSLC41A1-miR-9820-5p-SRSF1 axis regulates follicular granulosa cell apoptosis [85].

In humans, circRNAs have been profiled in granulosa cells in ovarian follicles [40], placenta [38,39,42,44,45,46,47,51,52,53,54,55,57,58,59,66,67,68,70,72,75,76,78,79,80,81,82,83,84], a multitude of different fetal tissues [36] and maternal blood [37,43,45,49,50,52,71,73,74,77,83], as well as exosomes isolated from umbilical cord blood [48,69]. Interestingly, one study confirmed that pregnancy-specific circRNAs were able to be detected in first-trimester platelets [49]. Many of these studies demonstrate differential circRNA expression profiles for disease states compared with an uncomplicated pregnancy, particularly comparisons in circRNA expression between PE, or gestational diabetes mellitus (GDM) and an uncomplicated pregnancy control. Some studies then went on to suggest particular circRNAs with biomarker potential for the pregnancy complication. Better studies followed this assertion by then performing functional studies to elucidate mechanisms of action for circRNAs of interest, utilising cell lines with circRNA overexpression or knockdown (data are presented in Table 1).

### 4.1. circRNAs in Preeclampsia

In placentae from women with PE, circ_0001438 [57], circ_0001687 [84], circ_0001855 [43], circ_0004904 [61], circ_0008726 [78], circ_0011460 [66], circ_0026552 [70], circ_0036877 [45], circ_0037078 [75], circ_0085296 [55], circ_0111277 [67], circ_101222 [37], circ_3286 [44], circBRAP [83], circLRRK1 [59], circSFXN1 [53], circTNRC18 [46] and circZDHHC20 [54] were elevated compared with uncomplicated pregnancy controls. A subset of these (circ_0001438, circ_0004904, circ_0008726, circ_0011460, circ_0026552, circ_0037078, circ_0085296, circ_0111277, circ_3286, circBRAP, circLRRK1, circSFXN1, circTNRC18 and circZDHHC20) when overexpressed in vitro resulted in decreased cell proliferation, migration, invasion or angiogenesis, or a combination of these effects. In contrast, circ_0001513 [84], circ_0007121 [68], circ_0017068 [82], circ_0032962 [76], circ_0051326 [77], circHIPK3 [80], circPAPPA [81], circ_PAPPA2 [79] and circUBAP2 [56] were decreased in PE placentae. A subset of these (circ_0007121, circ_0017068, circ_0032962, circHIPK3, circPAPPA and circUBAP2) when expressed in vitro promoted cell proliferation, migration, invasion or angiogenesis, or a combination of these effects. Many circRNAs studied were suggested to perform these functions through miRNA sponging but, as previously mentioned, the physiological impact of lowly expressed circRNAs sponging highly abundant miRNAs is debatable.

### 4.2. circRNAs in Gestational Diabetes Mellitus

Studies on placental circRNA expression in GDM focused mainly on profiling differences between GDM and uncomplicated pregnancies. In one study, first and early second-trimester maternal blood samples were collected to compare circRNA differential expression. These measures were then used to determine possible circRNA predictors for GDM development [50]. Other studies showed that circ_0008285 [60], circ_0026497 [71], circ_0039480 [71], circ-PNPT1 [63] and circVEGFC [74] were elevated in maternal plasma and whole blood from women with GDM. In vitro experiments using high glucose media for HTR-8/SVneo cell culture promoted proliferation and migration, which was reversed with circ_0008285 knockdown. Similarly, high glucose-induced arrest of cell viability and migration was reversed upon circ-PNPT1 knockdown. High levels of circVEGFC occurred with higher incidence rates of fetal malformation and hypertension. circ_0074673 [69] was upregulated in exosomes isolated from umbilical cord blood of GDM cases.

In contrast, other studies showed that circ_0001173 [60], circ_0005243 [52] and circ_102682 [73] were downregulated in placentae and maternal plasma from women with GDM. In vitro knockdown of circ_0005243 in HTR-8/SVneo trophoblast cells suppressed cell proliferation and migration, while circ_0001173 levels were positively correlated with glycated haemoglobin.

### 4.3. circRNAs in Other Pregnancy Complications

Other pregnancy complications have also been briefly studied with respect to circRNAs. One study reported almost 600 differentially expressed circRNAs in placentae from women with recurrent spontaneous abortion (RSA) compared with uncomplicated pregnancy [39]. Another study observed that circ_0050703 was downregulated in the placental villous tissue of patients with unexplained RSA (URSA), and circ_0050703 silencing in vivo reduced the number of successfully implanted embryos [64]. A circFOXP1/miR-143-3p/S100A11 axis was suggested in the RSA placentae [72]. Furthermore, circ-SETD2 was implicated in placental growth, with elevated circ-SETD2 in placentae of patients with fetal macrosomia [51]. In vivo overexpression experiments in HTR-8/SVneo cells showed increased cell proliferation and invasion. A circ_0074371/miR-582-3p/LRP6 axis was suggested in the context of fetal growth restriction [65]. Finally, granulosa cells from non-pregnant advanced age (≥38 years) compared with young age (≤30 years) women determined different circRNAs expression profiles depending on maternal age [40]. Whilst the number of studies into circRNAs in pregnancy is low, clearly circRNAs play many roles in pregnancy health, waiting to be discovered.

### 4.4. Limitations of circRNA Research

Research surrounding circRNAs in pregnancy is certainly still in its infancy. Several studies (Table 1) reported only the results of their profiling without any qPCR validation in independent samples. Whilst these data could be useful to other researchers, many of these profiling techniques have now been superseded with novel technologies. Methods such as RPAD [86], along with the use of Li^+^ ions in reaction buffers [87], in RNA-sequencing (RNA-seq) are proving much more reliable than the outdated circRNA arrays. circRNA detection through RNA-seq can be accurate assuming that one of these above methods is employed to prepare the samples.

Importantly, many of the studies described in this review lacked RNase R enrichment prior to sequencing. The addition of this exonuclease to a sample results in the digestion of all linear RNAs present, leaving an enriched population of circRNA transcripts. Not using this treatment prior to sequencing means that the depth of sequencing for circRNAs will be limited given that the sample is still composed primarily of linear RNA products. This likely results in the detection of only the most highly expressed circRNAs, leaving many transcripts undetected.

Finally, in studies using delivered placentae, authors should be cautious to declare specific circRNAs as causes of a pregnancy state. Given that these placentae have been collected after birth, without further mechanistic studies, it is unclear whether circRNAs in disease states are causal or an effect. However, these few studies provide enticing evidence to inspire further research into circRNAs in pregnancy and placental growth.

## 5. The Potential Importance of circRNAs in the Placenta: What We Can Apply from Our Knowledge of Cancer

The placenta can be considered a ‘controlled cancer’, as there are many parallels that have been previously drawn between placental development and cancer metastasis [88,89,90]. Some basic principles of cancer progression include tumour growth and tissue invasiveness (through epithelial to mesenchymal transition) [91], immune evasion and stimulation of angiogenesis [92], all of which are essential for successful placentation [93,94]. It is therefore unsurprising that many of the key molecular pathways are common to both placental development and cancer (Table 2). Importantly, the extensive research demand in the field of cancer has yielded a wealth of information about the molecular biology of cancers that can also be applied to the study of the placenta due to their many similarities.

Extensive mRNA profiling has been undertaken in placentae from the first trimester, second trimester and term [120,121,122,123]. Utilising this dataset shows that many of the genes responsible for circRNA production in cancer are also highly expressed in the placenta [124]. Hence, it is likely that circRNAs are also produced from these genes in the placenta. For example; the circRNA produced from *MYLK* has been shown to interfere with VEGFA signalling in bladder cancer [125]. The importance of the VEGF signalling pathway is well established in placentation [107], being most important for early angiogenesis and maintaining vascular health in the mother. Conversely, *MYLK* expression in the placenta increases across gestation. It is highly likely that circRNAs from the *MYLK* gene are also produced in the placenta and could impact the VEGF signalling pathway. There appear to be endless examples of genes which are known to produce circRNAs in cancer that are relevant to, and highly expressed in, placental development. We are currently profiling several of these circRNAs in the placenta and examining their roles in its development.

Importantly, circRNAs are not only able to affect development through their interactions with other molecules but they can also facilitate genomic instability through translocations. Rapid proliferation and consequent replication stress are common [126], both in cancer and in the placenta, resulting in possible DNA damage. Evidence has been provided for R-loop formation in plants, where a circRNA forms an RNA:DNA hybrid with its cognate DNA locus, stalling transcription and resulting in DNA breaks [127]. This was also shown to coincide with the recruitment of splicing factors, as well as alternative splicing. This genomic manipulation by circRNAs is likely to also occur in the eukaryotic tissues, although this is yet to be confirmed. If this is the case, dysregulation of circRNAs, particularly in early gestation, could result in genomic alterations that could affect both placental and fetal development and pregnancy health in general.

circRNAs have been shown to accumulate in a number of different tissues over time [128,129] and have been suggested to be a marker of tissue ageing. As the placenta is also known to undergo ageing [130], it is possible that circRNA accumulation in the placenta could occur. The implications of circRNA accumulation are still not well understood, but if these circRNAs continue to exert their functions as they accumulate this could lead to exaggerated circRNA action in the tissue.

## 6. Conclusions

Understanding the functions of circRNAs, particularly their involvement in placental development and pregnancy health, is in its infancy. However, these unique molecules are evidently the result of careful regulation, with multiple roles in physiological and pathophysiological conditions. Evidence that circRNAs may be involved in regulating placental development, and that differential circRNA profiles are found between healthy and complicated pregnancies, provides an imperative for further research.

## Figures and Tables

**Table 1:** Summary of research on circRNAs in female reproductive tissues and blood during pregnancy (limited to studies using primary tissue).

Author	Year	Tissue	Pregnancy Status	Key Findings	Limitations
Fan X, et al. [35]	2015	Mouse—oocytes and preimplantation embryos	Uncomplicated (assumed)	Detected 2891 circRNAs from 1316 host genes. A majority of these circRNAs are unique to the preimplantation stage and a large proportion of them exhibit dynamic expression patterns during this developmental process.	Sequencing using the SUPeR-seq method could possibly limit depth of circRNA sequencing due to no enrichment using RNase R.
Szabo L, et al. [36]	2015	Fetal tissue—unspecified, various species	Uncomplicated (assumed)	Developed an algorithm to compare established data sets in human, rat and mouse tissue and cell lines, with their generated circRNA data from RNA-seq on fetal tissue.	This study assumes a degree of conservation between species.
Zhang YG, et al. [37]	2016	Human maternal red blood cells	PE vs. uncomplicated	The levels of circ_101222 in red blood cells of patients with PE were significantly higher than in healthy women. Using ENG in combination with circ_101222 improved confidence for the prediction of PE.	
Qian Y, et al. [38]	2016	Human placenta	PE vs. preterm birth (PTB)	143 circRNAs were up-regulated and 158 were down-regulated in PE samples compared with preterm.	Use of microarray is not as comprehensive as sequencing techniques. PTB placenta is used as a gestational age control but PTB can occur for multiple reasons and is a pathology of pregnancy. Caution on interpretation is required.
Qian Y, et al. [39]	2017	Human placenta—chorionic villi	Recurrent spontaneous abortion (RSA) vs uncomplicated	594 aberrantly expressed circRNAs between gestational age-matched RSA and healthy placentae. Of these, 335 circRNAs were up-regulated and 259 were down-regulated.	No validation or investigation into results or mechanisms.
Cheng J, et al. [40]	2017	Human granulosa cells	Non-pregnant Advanced age (AA ≥ 38 years) vs. young age (YA ≤ 30 years)	46 upregulated and 11 downregulated circRNAs in AA samples compared with YA.	Use of microarray is not as thorough as sequencing techniques.
Zhang S, et al. [41]	2018	Mouse—endometrium	Uncomplicated (assumed)	Used microarray to find that 101 upregulated and 75 downregulated circRNAs at implantation sites compared with interimplantation sites. Four randomly selected circRNAs were validated for their expression using qRT-PCR	Use of microarray is not as comprehensive as sequencing techniques.
Bai Y, et al. [42]	2018	Human placenta	PE vs. uncomplicated	151 circRNAs were upregulated and 149 were downregulated in PE samples compared with normal. Possible biomarker hsa_circ_0007121 had a significant predictive index	Only 3 of the 10 circRNAs for validation matched sequencing results.
Jiang M, et al. [43]	2018	Human maternal peripheral blood mononuclear cells	PE vs. uncomplicated	884 circRNAs were downregulated and 1294 circRNAs were upregulated in PE samples compared with control. circ_0004904 and circ_0001855 combined with PAPP-A might be biomarkers for PE detection.	Use of microarray is not as thorough as sequencing techniques. As blood was collected at the time of disease, there is no way to be certain that circ_0004904 and circ_0001855 are causes of PE and not resultant.
Zhou W, et al. [44]	2018	Human placenta	PE vs. uncomplicated	Two circRNAs were up-regulated and 47 were down-regulated in PE compared with control placentae. Hsa_circRNA_3286 reduced invasion in HTR-8/SVneo cells.	RNase R enrichment for circRNAs not completed on RNA-seq samples. Only 3 of the 10 circRNAs for validation matched sequencing results.
Hu X, et al. [45]	2018	Human placenta Maternal whole blood	Severe PE vs. uncomplicated	4569 upregulated and 3984 downregulated circRNAs between severe PE and healthy pregnancy. Identified hsa_circ_0036877 as a potential novel blood biomarker for early PE.	Use of microarray is not as thorough as sequencing techniques. Significant differences in BMI, gestational age at delivery and % caesarean sections between severe PE and control groups.
Shen, XY, et al. [46]	2019	Human placenta	PE vs. uncomplicated	circTNRC18 upregulated in PE placentae. circTNRC18 reduced trophoblast cell migration and EMT. circTNRC18 repressed miR-762 activity and elevated Grhl2 protein.	
Wang H, et al. [47]	2019	Human placenta	GDM vs. uncomplicated	Three circRNAs were upregulated and 43 were downregulated between GDM and normal placentae. Ten randomly selected circRNAs were validated for their expression using qRT-PCR.	RNase R enrichment for circRNAs not completed on RNA-seq samples. Only 3 of the 10 circRNAs for validation matched sequencing results.
Cao M, et al. [48]	2020	Human umbilical cord blood	GDM vs. uncomplicated	229 upregulated and 278 downregulated circRNAs between GDM and healthy pregnancy. Exosome particle size was larger and exosome concentration was higher in GDM.	Use of microarray is not as thorough as sequencing techniques. RNase R enrichment for circRNAs not completed on microarray samples.
Oudejans C, et al. [49]	2020	Human maternal platelets	Uncomplicated (assumed)	Proof of concept study showing that pregnancy-specific circRNAs can be detected in first-trimester platelet RNA.	Could possibly limit depth of circRNA sequencing due to no enrichment using RNase R.
Yang H, et al. [50]	2020	Human maternal blood	GDM vs uncomplicated	Blood samples (n = 12) were collected from GDM and healthy pregnant women between 15–24 weeks’ gestation prior to RNase R treatment and circRNA microarray analysis.	Use of microarray for detection of circRNAs is not as thorough as sequencing techniques. No mention of gestational age matching between GDM and controls.
Wang D, et al. [51]	2020	Human placenta	Macrosomia vs. uncomplicated	Circ-SETD2 was upregulated in placentae of patients with fetal macrosomia. Circ-SETD2 upregulation increased proliferation and invasion in HTR-8/SVneo cells. Suggested circ-SETD2/miR-519a/PTEN axis involved in regulating trophoblasts in macrosomia.	Use of lnc-microarray for detection of circRNAs is not as thorough as sequencing techniques.
Wang H, et al. [52]	2020	Human placenta and maternal plasma	GDM vs. uncomplicated	Circ_0005243 was identified using RNase R (determining circular form). Circ_0005243 expression was downregulated in placenta and maternal plasma in GDM. Knockdown of circ_0005243 in HTR-8/SVneo cells suppressed cell proliferation and migration and increased secretion of inflammatory factors (TNF-α and IL-6). It also reduced β-catenin expression and increased nuclear NF-κB p65 nuclear translocation.	
Zhang Y, et al. [53]	2020	Human placenta and in vivo rat model	PE vs. uncomplicated	CircSFXN1 was identified using RNase R (determining circular form). CircSFXN1 was elevated in PE placenta. Knockdown of circSFXN1 promoted TEV-1 cell invasion and HUVEC angiogenesis—this effect was opposed with circSFXN1 overexpression. Pregnant rats injected with sFLT1-expressing adenovirus had in increased blood pressure and proteinuria; si-circSFXN1 reversed this. CircSFXN1 recruits sFLT1, validated by RNA-protein pulldown, RNA immunoprecipitation and dual-luciferase reporter assays.	Use of microarray is not as thorough as sequencing techniques, although the study validates these results with qRT-PCR.
Zhou B, et al. [54]	2020	Human placenta	PE vs. uncomplicated	CircZDHHC20 was identified using RNase R (determining circular form). CircZDHHC20 was up-regulated and miR-144 was down-regulated in PE placenta. CircZDHHC20 overexpression in HTR-8/SVneo cells repressed trophoblast proliferation, migration, and invasion. miR-144 regulated circZDHHC20 was inhibited by GRHL2.	
Zhu H, et al. [55]	2020	Human placenta	PE vs. uncomplicated	Circ_0085296 was identified using RNase R (determining circular form). Circ_0085296 elevated in PE placenta. Knockdown of circ_0085296 in HTR-8/SVneo cells promoted trophoblast cell proliferation, invasion, and migration. miR-144 down-regulated in PE placenta, directly bound to circ_0085296 and E-cadherin. Circ_0085296 bound to miR-144 to regulate E-cadherin.	
Qi T, et al. [56]	2020	Human placenta	PE vs. uncomplicated	CircUBAP2 (hsa_circ_0003496) was downregulated in PE placentae. CircUBAP2 knockdown suppressed HTR-8/SVneo cell proliferation and migration. CircUBAP2 sponges miR-1244 to regulate FOXM1. Cotransfection of si-circUBAP2 and a miR-1244 inhibitor partially restored cell proliferation and migration induced by circUBAP2 depletion.	
Li X, et al. [57]	2021	Human placenta	PE vs. uncomplicated	Circ_0001438 and NLRP3 were elevated in PE placenta. Knockdown of circ_0001438 promoted cell proliferation, migration and invasion but inhibited apoptosis and inflammatory responses in HTR-8/SVneo cells. Circ_0001438 bound to miR-942 to regulate NLRP3.	RNase R treatment not used—Circ_0001438 was not enriched for circular form only.
Ma B, et al. [58]	2021	Human placenta	PE vs. uncomplicated	252 upregulated and 109 downregulated circRNAs between preeclamptic and healthy placentae; 6 circRNAs were further validated using qPCR.	No mention of gestational age matching between PE and controls.
Tang R, et al. [59]	2021	Human placenta	PE vs. uncomplicated	CircLRRK1 was identified using Actinomycin D and RNase R (determining circular form). circLRRK1 was elevated in PE placenta. Knockdown of circLRRK1 promoted cell proliferation, migration and invasion in HTR-8/SVneo cells. circLRRK1 bound to miR-223-3p to regulate PI3K/Akt signalling.	
Chen H, et al. [60]	2021	Maternal plasma	GDM vs. uncomplicated	Circ_0008285 was increased, while circ_0001173 was decreased, in GDM samples. Circ_0008285 correlated with total cholesterol and LDL-C levels. Circ_0001173 correlated with glycated haemoglobin. High glucose media promoted HTR-8/SVneo cell proliferation, invasion, and migration, while circ_0008285 knockdown exerted the opposite effect.	High glucose media contained 30 mmol/L glucose which is potentially too high for physiological relevance.
Dai W, et al. [61]	2021	Human placenta and maternal plasma	PE vs. uncomplicated	Circ_0004904 levels were elevated in PE placentae and maternal plasma. Aberrant circ_0004904 expression inhibited autophagy and induced JEG3 cell proliferation and invasion. Circ_0004904 also regulated ATG12 levels via miR-570, as well as controlling the FUS/VEGF axis in HTR-8/SVneo and JEG3 cells.	
Ping Z, et al. [62]	2021	Maternal blood	PE vs. uncomplicated	121 differentially expressed circRNAs were upregulated and 30 downregulated in PE samples. Functional and pathway enrichment analysis was conducted using Gene Ontology and KEGG databases.	Could possibly limit depth of circRNA sequencing due to no enrichment using RNase R. No mechanistic studies conducted or PCR validation of results.
Zhang L, et al. [63]	2021	Human placenta	GDM vs. uncomplicated	Circ-PNPT1 levels were elevated in GDM placentae and high glucose (HG)-induced HTR-8/SVneo cells. HG-induced arrest of cell viability, migration, invasion and apoptosis was reversed with circ-PNPT1 knockdown. Circ-PNPT1 also sponged miR-889-3p to regulate PAK1. HTR-8/SVneo cell line experiments showed circ-PNPT1 was packaged into exosomes and internalised by surrounding cells.	Exosome studies were conducted in HTR-8/SVneo cell line, not replicated in placental explants or maternal blood. High glucose media contained 25 mmol/L glucose which is potentially too high for physiological relevance.
Tang M, et al. [64]	2021	Human placental villous tissue and embryos In vivo mouse model	Unexplained recurrent spontaneous abortion (URSA) vs. uncomplicated	Circ-0050703 (circRNA-DURSA) is downregulated in URSA placental villous tissue. In vitro, circRNA-DURSA silencing results in cell apoptosis and circRNA-DURSA competitively binds miR-760, regulating HIST1H2BE. In vivo, circRNA-DURSA silencing decreased number of embryos successfully implanted.	
Yao P, et al. [65]	2021	Human placenta	Fetal growth restriction vs. uncomplicated	Circ_0074371 and LRP6 were downregulated, and miR-582-3p was upregulated in fetal growth restriction (FGR) placentae and HTR-8/SVneo cells. Circ_0074371 sponges miR-582-3p to regulate LRP6. Circ_0074371 knockdown induced HTR-8/SVneo cell cycle arrest, apoptosis, and inhibited cell proliferation, migration, and invasion, which was reversed with a miR-582-3p inhibitor.	
Fan Z, et al. [66]	2021	Human placenta	PE vs. uncomplicated	Circ_0011460 is upregulated in PE placentae, and overexpression in HTR-8/SVneo cells suppressed proliferation, migration and invasion, and increased cell apoptosis. Circ_0011460 also sponges miR-762 and regulates HTRA1.	
Li C, et al. [67]	2021	Human placenta	PE vs. uncomplicated	Circ_0111277 and NFAT5 expression were increased in PE placentae and miR-424-5p was decreased. Circ_0111277 knockdown increased cell viability, migration, invasion, and angiogenesis in HTR-8/SVneo cells. Circ_0111277 acted as a sponge of miR-424-5p to regulate NFAT5 expression.	
Zhou F, et al. [68]	2022	Human placenta	PE vs. uncomplicated	Circ_0007121 is downregulated in PE placentae. Upregulation of circ_0007121 promotes cell proliferation, migration, invasion and EMT. Circ_0007121 also sponges miR-421 and thus regulates ZEB1 expression.	
Huang Y, et al. [69]	2021	Human umbilical cord blood—exosomes isolated	GDM vs. uncomplicated	Larger exosomes and greater number of exosomes in umbilical cord blood of GDM patients. Circ_0074673 was upregulated in exosomes from GDM and in HUVECs co-cultured with exosomes. Loss of exosomal circ_0074673 facilitated the proliferation, migration, and angiogenesis of high glucose-HUVECs via the miR- 1200/MEOX2 axis.	High glucose media contained 25 mM glucose which is potentially too high for physiological relevance.
Shan L, et al. [70]	2021	Human placenta	PE vs. uncomplicated	miR-331-3p negatively correlates with circ_0026552 relative expression, while TGF-βR1 positively correlates with circ_0026552 expression. Silencing circ_0026552 increased proliferation, migration and invasion of HTR-8/SVneo cells, which was reversed with circ_0026552 overexpression. Circ_0026552 sponges miR-331-3p to upregulate TGF-βR1 expression.	RNase R enrichment for circRNAs not completed on microarray samples. Use of microarray is not as thorough as sequencing techniques, although the study validates these results with qRT-PCR. Small number (n = 3 PE, n = 4 control) tissues used for microarray.
Jiang B, et al. [71]	2021	Maternal blood	GDM vs. uncomplicated	Plasma exosomal circRNA_0039480 and circRNA_0026497 were increased in GDM. circRNA_0039480 was elevated in GDM vs. normal glucose tolerance control throughout trimesters and positively correlated with OGTT during the second trimester. The combination of circRNA_0039480 and circRNA_0026497 suggested as a useful biomarker for GDM in the first trimester (AUC = 0.754, *p* < 0.001).	RNase R enrichment for circRNAs not completed on microarray samples. Use of microarray is not as thorough as sequencing techniques, although the study validates these results with qRT-PCR. Small number (n = 3) samples used for microarray.
Gao Y, et al. [72]	2021	Human placenta	Recurrent pregnancy loss (RPL) vs. uncomplicated	MiR–143–3p targeted S100A11 and was negatively regulated by circFOXP1. miR–143–3p competitively bound circFOXP1. circFOXP1 regulated HTR-8/SVneo cell functions through the miR–143–3p/S100A11 axis.	
Wu H, et al. [73]	2022	Maternal blood	GDM vs. uncomplicated	Circ_102682 was decreased in GDM blood samples. circRNA_102682 was significantly correlated with triglycerides, APOA1, APOB, 1-h blood glucose in the serum of GDM patients.	
She W, et al. [74]	2021	Maternal plasma	GDM vs. uncomplicated	CircVEGFC regulates glucose metabolism—higher incidence of GDM in patients with high circVEGFC levels. Elevated circVEGFC levels in GDM plasma. High circVEGFC level group showed higher incidence rates of fetal malformation and hypertension.	Correlation established but further mechanistic studies required to establish whether this is causative.
Zou H, et al. [75]	2022	Human placenta	PE vs. uncomplicated	Circ_0037078 is upregulated in PE placentae. Knockdown of circ_0037078 increases trophoblast cell proliferation, migration, invasion and angiogenesis. Circ_0037078 also sponges miR-576-5p and increases IL1RAP expression.	
Mao Q, et al. [76]	2021	Human placenta	PE vs. uncomplicated	Circ_0032962 and PBX3 levels were decreased in PE placentae and miR-326 was elevated. Circ_0032962 knockdown suppressed cell proliferation ability, migration, invasion, and EMT in HTR-8/SVneo cells.	
Wang L, et al. [77]	2021	Maternal blood	PE vs. uncomplicated	Circ_0051326 and HLA-G protein and mRNA were decreased in PE samples. There was a positive correlation between the expression of serum circ_0051326 with HLA-G mRNA.	
Shu C, et al. [78]	2021	Human placenta	PE vs. uncomplicated	Silencing circ_0008726 promoted cell migration and EMT, while circ_0008726 overexpression suppressed these processes. Circ_0008726 sponged miR-345-3p to regulate RYBP expression. Circ_0008726 was negatively correlated with miR-345-3p and positively correlated with RYBP expression levels in PE placentae. Transfection of miR-345-3p mimic or RYBP knockdown counteracted the effects of circ_0008726 overexpression on cell migration and EMT.	
Zhang Y, et al. [79]	2021	Human placenta	PE vs. uncomplicated	Increases in m6A-modified circRNAs are prevalent in PE placentae, with the main methylation changes occurring in the 3′ UTR and near the start codon. In PE, circPAPPA2 levels are decreased while m6A modification is increased. METTL14 increases circPAPPA2 m6A methylation and IGF2BP3 maintains circPAPPA2 stability.	
Wang W, et al. [80]	2021	Human placenta	PE vs. uncomplicated	In PE placentae, circHIPK3 and KCMF1 were downregulated and miR-346 was upregulated. CircHIPK3 overexpression promotes trophoblast cell proliferation, migration and invasion, as well as decreasing cell cycle arrest and apoptosis. CircHIPK3 also targets miR-346 and regulates KCMF1 expression.	
Li J, et al. [81]	2022	Human placenta	PE vs. uncomplicated	CircPAPPA positively regulates trophoblast cell proliferation, migration and invasion, and causes apoptosis and cell cycle arrest, through the miR-3127-5p/HOXA7 axis.	
Wang W, et al. [82]	2022	Human placenta	PE vs. uncomplicated	Circ_0017068 was downregulated in PE placental samples. Circ_0017068 overexpression promoted HTR-8/SVneo cell proliferation, cycle progression, and suppressed apoptosis while silencing of circ_0017068 exhibited opposite effects. Circ_0017068 targeted miR-330-5p to regulate XIAP expression, and through this regulated proliferation, cycle progression, and apoptosis.	
Zhang Y, et al. [83]	2021	Human placenta and maternal plasma	PE vs. uncomplicated	PE predictive power was greatest when plasma sFLT1 and circBRAP levels were combined with uterine pulsatility index. CircBRAP was increased in PE placentae and may regulate miR-106b to decrease TEV-1 cell proliferation, invasion and apoptosis.	
Yuan Y, et al. [84]	2022	Human placenta	PE vs. uncomplicated	2432 circRNAs were differentially expressed between PE and control tissues. hsa_circRNA_0001687/hsa-miR-532-3p/MMP14/AXL, hsa_circ_0001513/hsa-miR-188-5p/HMGCS1 and hsa_circ_0001513/hsa_circ_0001329/hsa-miR-760/MAP1LC3B axes may contribute to PE pathogenesis.	The paper uses publicly available datasets instead of completing their own sequencing. As such, data quality cannot be ascertained. No luciferase assays to validate the potential axes listed.
Wang H, et al. [85]	2022	Pig ovaries	Atretic vs. healthy follicles	CircSCL41A1 was elevated in healthy follicles compared with atretic follicles. miR-9820-5p competitively binds circSLC41A1 to regulate SRSF1. A circSLC41A1-miR-9820-5p-SRSF1 axis regulates follicular granulosa cell apoptosis.	

**Table 2:** Key signalling pathways common to placental development and cancer, with references.

Signalling Pathway	Placenta	Cancer
EGFR	[95]	[96]
PI3K/Akt	[97]	[98]
P53	[99]	[100]
IGF	[101,102]	[103,104]
TGF-β	[105]	[106]
VEGF	[107]	[108]
mTOR	[109,110]	[111]
MAPK	[112]	[113]
Jak/STAT	[114]	[115]
Wnt/β-catenin	[116]	[117]
NF-κB	[118]	[119]

## Data Availability

Not applicable.
